# Effects of extreme thermal conditions on plasticity in breeding phenology and double-broodedness of Great Tits and Blue Tits in central Poland in 2013 and 2014

**DOI:** 10.1007/s00484-016-1152-9

**Published:** 2016-03-17

**Authors:** Michał Glądalski, Mirosława Bańbura, Adam Kaliński, Marcin Markowski, Joanna Skwarska, Jarosław Wawrzyniak, Piotr Zieliński, Jerzy Bańbura

**Affiliations:** 1 Department of Experimental Zoology and Evolutionary Biology, Faculty of Biology and Environmental Protection, University of Łódź, Banacha 12/16, 90-237 Łódź, Poland; 2 Museum of Natural History, Faculty of Biology and Environmental Protection, University of Łódź, Kilińskiego 101, 90-011 Łódź, Poland; 3 Department of Teacher Training and Biological Diversity Studies, Faculty of Biology and Environmental Protection, University of Łódź, Banacha 1/3, 90-237 Łódź, Poland; 4 Department of Ecology and Vertebrate Zoology, Faculty of Biology and Environmental Protection, University of Łódź, Banacha 12/16, 90-237 Łódź, Poland

**Keywords:** *Parus major*, *Cyanistes caeruleus*, Climate change, Climate warming, Laying date, Second clutch, Extreme weather event, Delayed breeding time, Accelerated breeding time

## Abstract

Many avian species in Europe breed earlier as a result of higher temperatures caused by global climate changes. Climate change means not only higher temperatures but also more frequent extreme weather events, sometimes contrasting with the long-term trends. It was suggested that we should look closely at every extreme phenomenon and its consequences for the phenology of organisms. Examining the limits of phenotypic plasticity may be an important goal for future research. Extremely low spring temperatures in 2013 (coldest spring in 40 years) resulted in birds laying unusually late, and it was followed in 2014 by the earliest breeding season on record (warmest spring in 40 years). Here, we present results concerning breeding phenology and double-broodedness in the Great Tit (*Parus major*) and the Blue Tit (*Cyanistes caeruleus*) in 2013 and 2014 in an urban parkland and a deciduous forest in central Poland. Great Tits started laying eggs 18.2 days later in 2013 than in 2014 in the parkland, whereas the analogous difference was 21.1 days in the forest. Blue Tits started laying eggs in the parkland 18.5 days later in 2013 than in 2014, while the analogous difference was 21.6 days in the forest. The difference in the proportion of second clutches in Great Tits between 2013 (fewer second clutches) and 2014 (more second clutches) was highly significant in the parkland and in the forest. This rather large extent of breeding plasticity has developed in reaction to challenges of irregular inter-annual variability of climatic conditions. Such a buffer of plasticity may be sufficient for Blue Tits and Great Tits to adjust the timing of breeding to the upcoming climate changes.

## Introduction

The impact of climate changes on populations of birds has recently been under extensive study (Mitrus et al. 2005; Potti 2009; Wesołowski and Cholewa 2009; Goodenough et al. 2011, 2015; Chmielewski et al. 2013; Whitehouse et al. 2013). It has been shown that many avian species in Europe breed earlier as a result of higher temperatures caused by global climate changes (Both and Visser 2005; Wesołowski and Cholewa 2009; Bauer et al. 2010; Matthysen et al. 2010; Fletcher et al. 2013; Bartošová et al. 2014). It has also been suggested that climate change not only means higher temperatures but also more frequent extreme weather events (Tebaldi et al. 2007; Coumou and Rahmstorf 2012; McCarthy et al. 2012; Zhang et al. 2012; Tang et al. 2013; Richter 2015; Feser et al. 2015). Extreme weather events are likely to disturb life history strategies of some species and make it more difficult for organisms to adapt to local environments (Visser 2008; Chamberlain and Pearce-Higgins 2013; Tobolka et al. 2015; Indykiewicz 2015). On the other hand, it was suggested that the recent extreme weather events can be treated as a natural experiment that may elucidate the mechanisms by which birds adjust their phenology to fluctuating environments (Wesołowski et al. 2016). The question is whether current changes in the timing of breeding can be attributed to evolutionary changes in response to the documented selection pressures, or whether they result from individual plasticity—the capacity of an individual to fit its phenology to environmental conditions (Charmantier et al. 2008; Piersma and Gils 2011; Charmantier and Gienapp 2014). If phenotypic plasticity plays a major role in adjusting to unpredictable weather conditions in spring, examining the limits of plasticity may be an important goal for future research (Charmantier et al. 2008; Piersma and Gils 2011; Stamps 2015; Wesołowski et al. 2016).

Maximizing the number of broods raised during a single breeding season could be a good reproductive strategy if it does not cause a decrease in the rates of survival of parents or produced young (Williams 1966; Stearns 1992). The Great Tit (*Parus major*) is an optionally double-brooded species—only some pairs produce a second clutch after successfully fledging young from the first brood (Gosler 1993). High variation in the frequency of second broods between breeding seasons is commonly observed in this species (Lack 1958; Mägi and Mänd 2004). Verboven and Verhulst (1996) showed that the timing of breeding is the most crucial factor influencing the decision to have or not to have a second brood in the current breeding season.

**Fig. 1:**
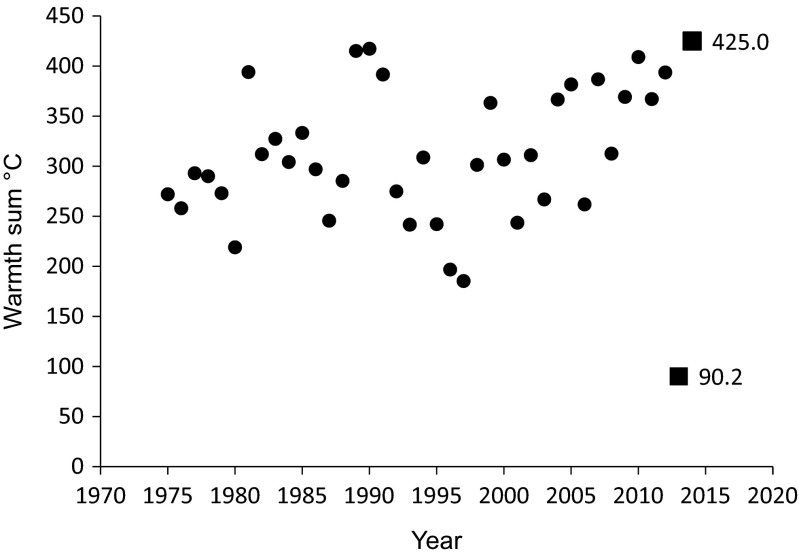
Warmth sums (sum of the daily maximum temperatures in the period of 15 March–15 April) by year for the period of 1975–2014 (the most extreme 2013, 90.2 °C, and 2014, 425 °C, were marked as *squares*)

Temperature has a major influence on the optimal laying date of insectivorous passerines. It affects the timing of peaks in spring caterpillar abundance (Perrins 1991; Thorley and Lord 2015). In 2013, low spring temperatures caused birds to lay unusually late (Glądalski et al. 2014), and it was followed by the earliest breeding season on record in 2014 (temperature characteristics of that period showed that those 2 years were the most extreme in 40 years; Fig. 1).

In this paper, we report on responses of Great Tits and Blue Tits (*Cyanistes caeruleus*) to extreme spring temperature variation between 2013 and 2014. The data were gathered in two different habitats (an urban parkland and a deciduous forest), in Łódź (central Poland), within an ongoing long-term study into breeding biology of hole-nesting birds occupying nestboxes (Banbura et al. 2013; Glądalski et al. 2015). We predict that laying dates should depend on ambient temperature in both tit species and that an early breeding season should induce Great Tits to have more second clutches (as early laying females tend to have more second clutches) and vice versa.

## Material and methods

This study was conducted in 2013 and 2014 as part of a long-term research project (started in 1999; Glądalski et al. 2015), concerning the breeding biology of secondary hole-nesting birds occupying nestboxes near Łódź, central Poland (51° 47′ N, 19° 28′ E). The study sites are located in two floristically and structurally contrasting habitats, an urban parkland (51° 45′ N, 19° 24′ E) and a deciduous forest (51° 50′ N, 19° 29′ E), 10 km apart. The urban parkland area (80 ha) consists of the zoological garden (16 ha) and the botanical garden (64 ha). This area is one of the biggest recreation and entertainment areas in Łódź (Glądalski et at. 2016a). It has a highly fragmented tree cover, formed artificially with large areas of tree-free spaces. The vegetation mainly consists of hornbeam *Carpinus betulus*, pedunculate oak *Quercus robur*, Scots pine *Pinus silvestris*, birches *Betula* sp., poplars *Populus* sp., willows *Salix* sp., limes *Tilia* sp., maples *Acer* sp., and many exotic tree species. The forest study site (130 ha) is situated in the Łagiewniki forest—a rich deciduous forest, located north of Łódź. Large parts of the forest come directly from the ancient woodland typical for this region of central Europe. Oaks (*Q. robur* and *Q. petraea*) are the dominant tree species.

The study sites were supplied with standard wooden nestboxes (Lambrechts et al. 2010) with a removable front wall and with internal dimensions of 11.5 (depth) × 11 (width) × 30 (height) cm and a 3-cm-diameter entrance located 20 cm from the bottom of the nestbox (ca. 200 nestboxes were set in the parkland and ca. 300 nestboxes were set in the forest). Second clutches are clutches produced by females that had a successful first clutch (with at least one fledged young) in the same year. A total of 227 first clutches and 42-s clutches of the Great Tit were studied in 2013 and 2014. A total of 80 first clutches of the Blue Tit were studied in 2013 and 2014 (there were no second clutches in Blue Tits).

The local temperatures for Łódź were obtained from TuTiempo.net database (http://en.tutiempo.net/climate/ws-124650.html). As an indicator of thermal conditions for tits, we used a pre-laying-early-laying warmth sums of the daily maximum temperatures between 15 March and 15 April each year (Glądalski et al. 2014, 2015; Wawrzyniak et al. 2015). The mean laying dates are described as days from 1 March.

We used ANOVA to compare mean laying dates between years and study areas. Fisher’s exact test was used to test for differences in the proportion of second clutches between extreme years in the parkland and in the forest. Graphical and statistical analyses were performed using STATISTICA 10 (StatSoft Inc. 2011).

## Results

The patterns of variation in temperature were strikingly different between the study years (Table 1). In 2013, temperatures below zero were prevailing in March and at the beginning of April. The snow cover started to disappear in mid-April, and in the parkland, the snow melted at least 1 week before the forest (Glądalski, personal observations). In 2014, there was no snow cover during March and April and the temperatures were much higher than in the previous year (Table 1). The warmth sum in 2013 was the lowest in 40 years (1975–2014), 90.2 °C (the second coldest was in 1997, 185.3 °C), whereas the warmth sum in 2014 was the highest in 40 years, 425.0 °C (the second warmest was in 1990, 417.3 °C; Fig. 1).

**Table 1:** Mean temperature (average of mean daily temperatures) and mean maximum temperature (average of maximum daily temperatures) in parentheses for three periods of spring and the warmth sum for 15 March–15 April (the sum of the daily maximum temperatures during the period of 15 March–15 April)

Variable	2013—Cold	2014—Warm
Mean temperature °C (mean max. temp. °C)		
1–15 March	−0.9 (2.8)	4.6 (10.1)
16–31 March	−3.9 (−0.3)	8.3 (13.0)
1–15 April	3.2 (6.4)	7.8 (13.9)
Warmth sum (°C)		
15 March–15 April	90.2	425.0

**Fig. 2:**
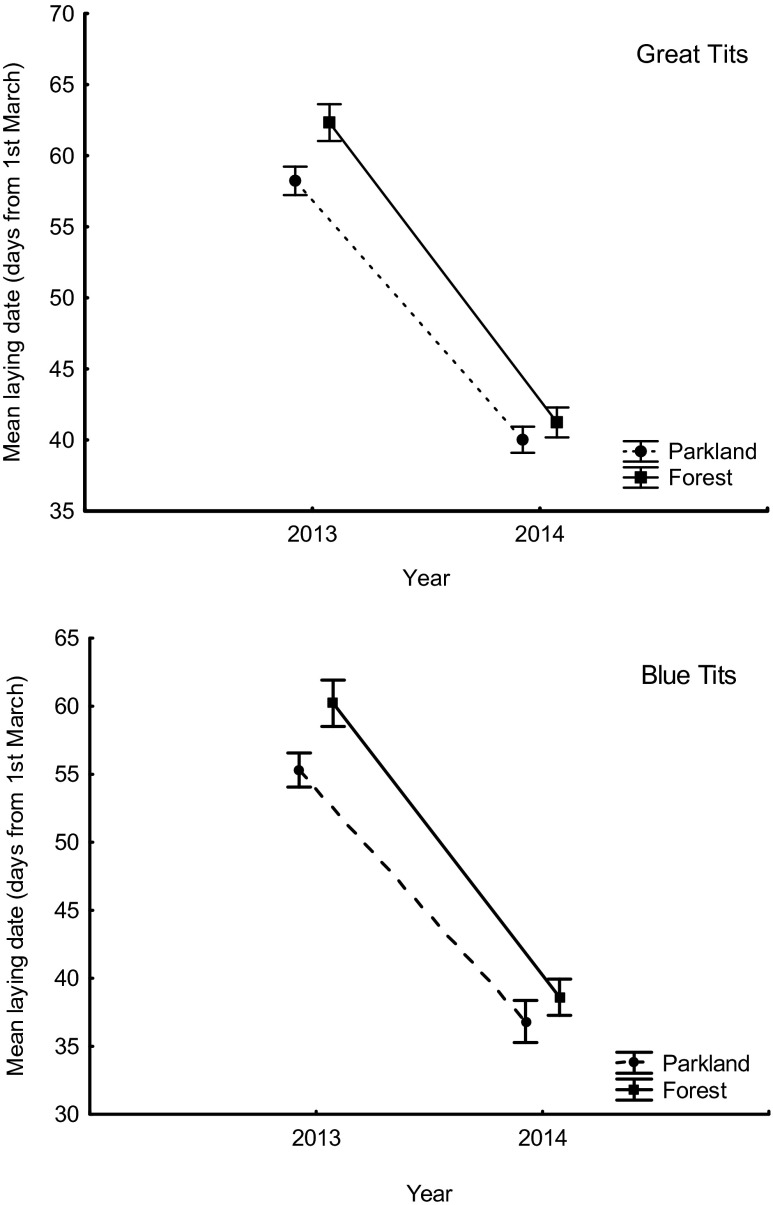
Mean laying dates (1 = 1 March) in parkland and forest areas in Great Tits and Blue Tits in the extreme years of 2013 and 2014. Mean laying dates are represented as averages ± 95 % confidence intervals

Great Tits started laying eggs much later in 2013, mean for the parkland 27 April (58.2 days from 1 March) and mean for the forest 1 May (62.3 days from 1 March), and very early in 2014, mean for the parkland 9 April (40.0 days from 1 March) and mean for the forest 10 April (41.2 days from 1 March). Blue Tits also started laying eggs late in 2013, mean for the parkland 24 April (55.3 days from 1 March) and mean for the forest 29 April (60.2 days from 1 March), and very early in 2014, mean for the parkland 6 April (36.8 days from 1 March) and mean for the forest 8 April (38.6 days from 1 March; Fig. 2). The difference between years in Great Tits was highly significant, for the parkland *F*_1,123_ = 602.2, *p* < 0.001 and for the forest *F*_1,83_ = 812.6, *p* < 0.001 (Fig. 2). The difference between years in Blue Tits was also highly significant, for the parkland *F*_1,41_ = 318.6, *p* < 0.001 and for the forest *F*_1,35_ = 433.7, *p* < 0.001 (Fig. 2). The mean difference in the Great Tit laying date between 2013 and 2014 was 18.2 days for the parkland and 21.1 days for the forest. The mean difference in the Blue Tit laying date between the years was 18.5 days for the parkland and 21.6 days for the forest. In 2013, the earliest individual female Great Tit started to lay on 18 April in the parkland (in 2014, it was 1 April) and on 19 April in the forest (in 2014, it was 2 April). The earliest individual Blue Tit started to lay on 20 April 2013 in the parkland (in 2014, it was 1 April) and on 27 April 2013 in the forest (in 2014, it was 3 April).

The influence of the year × habitat type interaction on laying date in Great Tits was significant (*F*_1,206_ = 6.9, *p* = 0.009), suggesting that the urban parkland advantage over forest was more apparent in 2013 (Fig. 2).The significant effect of the year × habitat type interaction on laying date in Blue Tits (*F*_1,76_ = 4.5, *p* = 0.038) suggested that the difference between mean laying dates at study areas in 2013 was significant, while there was no difference in 2014 (Fig. 2).

Only 6-s clutches in 2013 and as many as 36-s clutches in 2014 were produced by Great Tits in both the study sites. The difference in the proportion of second clutches between 2013 and 2014 was highly significant in the parkland and in the forest and overall (Table 2).

**Table 2:** Number of first clutches and second clutches of Great Tits in 2013 and 2014 at both study areas

Year	2013	2014	Fisher’s exact test, *p*
Parkland first clutches	75	68	0.001
Parkland second clutches	6	25	
Forest first clutches	34	50	<0.007
Forest second clutches	0	11	
Sum first clutches	109	118	<0.001
Sum second clutches	6	36	

Fisher’s exact test examines differences in the proportion of second clutches between years in the parkland and in the forest and overall, *p* ≤ 0.05 was considered as significant

## Discussion

In the present study, we investigated variation in laying date in two tit species, the Great Tit and the Blue Tit, in two extremely different, subsequent years (2013 and 2014), showing that laying date is a highly plastic trait that is strongly dependent on spring temperature. Because higher latitudes have larger climatic variation than lower latitudes, natural selection should promote broader climatic adaptability and thermal tolerance in individuals at higher latitudes as postulated by the climatic variability hypothesis (Allee et al. 1949). This hypothesis may be considered in the context of avian breeding phenology and recent climate changes. The birds we studied adjusted their laying dates in accordance with changes in spring temperature, thus corroborating results from other tit populations (Charmantier et al. 2008; Thorley and Lord 2015; Wesołowski et al. 2016).

Timing of the breeding in tits is clearly correlated with the ambient temperature prior to breeding initiation (van Balen 1973; Perrins and McCleery 1989). Higher temperature directly reduces energy costs associated with thermoregulation and causes faster growth of gonads and formation of eggs. The leafing phenology of trees directly influences the occurrence of the most important component of the diet of chicks—caterpillars (Bańbura et al. 1999). Glądalski et al. (2014) previously showed that the mean date of the first laid egg in Great Tits and Blue Tits was highly negatively correlated with the warmth sums in the parkland and in the forest areas. Two consecutive breeding seasons analyzed in this paper demonstrated large flexibility in decisions about when to start reproduction in both species of tits (e.g., the difference between first laid egg in 2013 and 2014 in one individual Great Tit female was 24 days; unpublished data). Tits were able to shift their timing of egg laying by about 3 weeks between 2 years. Similar extremes in the same years were recorded in the Marsh Tit (*Poecile palustris*) in Białowieża (eastern Poland) by Wesołowski et al. (2016). Wesołowski et al. (2016) showed that individual March Tits from Białowieża were able to shift their timing of breeding more than 3 weeks between 2013 and 2014. Those authors suggest that large inter-annual variation of breeding dates that they observed in Białowieża (see also Wesołowski and Cholewa 2009) could be accounted for by individual plasticity, and invoking other mechanisms would be redundant. Our present results confirm the existence of large plasticity in the timing of laying in Great Tits and Blue Tits. It seems probable that even if springs proved to be still warmer in the future (as climatologists predict), tits would be already prepared—they posses physiological and behavioral mechanisms that allow them to adjust to such a challenge, as suggested by Wesołowski et al. (2016).

The later initiation of breeding by tits in forest areas compared to nearby urban areas was shown by many authors (Lack 1958; Dhondt et al. 1984; Cowie and Hinsley 1987; Glądalski et al. 2015; Wawrzyniak et al. 2015). Early breeding in urban populations was shown to be caused mainly by food availability. Human-provided food may induce earlier laying through improving the body condition of females (Chamberlain et al. 2009). On the other hand, taxonomic composition of tree flora in our study parkland may result in earlier leafing as suggested by Marciniak et al. (2007). Buds and, then, larvae on oaks in the forest appear later than on birches and poplars in the parkland. In 2014 (warm, early breeding season), there was no snow cover, the temperatures were relatively high during March, and the conditions for breeding were favorable. In 2013 (cold, late breeding season), the snow cover was present until mid-April, but in the parkland, the snow melted at least 1 week before the forest. This could also lead to a difference in the abundance of food for females. Probably, urban heat island effects partly influenced the phenology of urban population of tits (Rebele 1994). The lack of sufficient food for females restricts their early breeding (Perrins 1970; Nager and van Noordwijk 1995). Additionally, because snail shells are the main source of calcium necessary for females to form the shells of their eggs (Bańbura et al. 2010; Reynolds and Perrins 2010), snow cover may make it more difficult to find them. A tit nest consists of moss layer (sometimes with dry grass and roots) and the lining layer (animal material including wool, silk, fur, feathers, or hair; Glądalski et al. 2016b). Snow and low temperatures can also make it difficult to obtain the material to build nests and, in this way, may delay breeding (Britt and Deeming 2011; Deeming et al. 2012; Wawrzyniak, unpublished data).

The timing of breeding was shown to be the most crucial factor influencing the decision on having a second brood in the current breeding season in Great Tits (Verboven and Verhulst 1996). The frequency of second clutches was also shown to be dependent on habitat. Because the period of peak caterpillar abundance is short and food availability notably declines after the first brood in deciduous forests, second clutches are less frequent there than in urban and suburban green spaces, where food availability remains more stable during the entire breeding season (Lack 1958; Gosler 1993; Mägi and Mänd 2004). Orell and Ojanen (1983a, 1983b) showed that female tits that decided to have a second clutch produced their first clutch earlier than females that did not have a second clutch. The natural experiment described in the present paper shows that Great Tits tend to have second clutches when the breeding season starts early (warm spring, 2014), and they tend to avoid second clutches when the breeding season starts late (cold spring, 2013).

In conclusion, the breeding date of Great Tits and Blue Tits turned out to be a flexible trait. Populations of both tit species may tune their egg-laying dates to diverse weather conditions by about 3 weeks. As suggested by Wesołowski et al. (2016), this rather large range of plasticity has developed in reaction to challenges imposed by irregular inter-annual climatic variation. It should suffice to adjust bird’s breeding decisions to the forecasted climate changes. Further investigations should focus on the impact of extreme events on adjustment of tits to match breeding with timing of peaks in spring caterpillar abundance across the latitudes.
